# Supplementary material for: Changes in dietary habit and physical activity among the diabetic patients of Bangladesh during COVID-19: A cross-sectional study
Source: PLOS Glob Public Health. 2023 Jun 29;3(6):e0002096. doi: 10.1371/journal.pgph.0002096 (PMC10309609; doi:10.1371/journal.pgph.0002096)
Supplement: Attachment — Submitted filename: Response to Reviewers.docx [file pgph.0002096.s002.docx]

May 15, 2023

Dear Editor,

First, we would like to thank you for considering our work in your prestigious journal.

Second, we took your and the reviewer’s comment seriously and worked extensively to improve the quality of the article. Below you will find a point-by-point explanation/modification/addition *in italic* of the suggestions/recommendations provided by the reviewer.

Finally, according to the reviewer’s recommendation, we have completely re-analyzed the change pattern of the dietary habits and physical activity using the appropriate McNemar-Bowker test.

Should you have any other questions or concerns, please do not hesitate to contact us.

Sincerely,

Dr. Azaz Bin Sharif

(On behalf of the research team)

**Journal Requirements:**

1. Please send a completed 'Competing Interests' statement, including any COIs declared by your co-authors. If you have no competing interests to declare, please state "The authors have declared that no competing interests exist". Otherwise please declare all competing interests beginning with the statement "I have read the journal's policy and the authors of this manuscript have the following competing interests:"

*We’ve declared the ‘Competing Interests’ statements on line 398 of the clean version of revised manuscript.*

2. If you did not receive any funding for this study, please simply state: “The authors received no specific funding for this work.”

*We’ve stated the ‘Funding’ statement on line 400 of the clean version of revised manuscript.*

**Additional Editor's Comments to Author/s**:

The study is appropriate and relevant but there are revisions suggested by the reviewers which I am emphasizing that the authors pay particular attention to.

**Abstract:** The abstract to this study needs to be revised to communicate adequately what the study sought to achieve and what was found. The following represent revised suggestions in the abstract:

Lines 17-25: “In an effort to avert further Covid-19 transmission, the Bangladesh government took several initiatives which disrupted the routine food intake and exercise of diabetic patients. This study sought to examine the difference in dietary and exercise habits these patients between their pre-pandemic status and during COVID-19 which may be attributed to the observed poor health outcomes during the study period.

*We have carefully considered your recommendations and have revised the abstract accordingly. We agree that the suggested changes provide a clearer understanding of the study's objectives and findings. The revised abstract now reads according to your suggestion in lines 18-22 of the clean version. We believe, the revised abstract better communicates the purpose of our study and its results. We hope these changes address your concerns and improved the overall quality of our article.*

**Methods:** This was a cross-sectional study design that enrolled 604 confirmed patients with type- I and type-2 diabetes Mellitus by convenience sampling method attending outpatient clinics in three hospitals in Bangladesh. A validated semi-structured questionnaire was developed, using the Kobo toolbox, to collect information by interviewer-administered technique regarding self-reported eating habit and physical activities of patients before and after COVID-19.”
The results and conclusion need to align with the revised results. The results suggested other factors are involved in the study but are not mentioned.

*The method section of the abstract has been revised as suggested. As you pointed out, the results suggested the involvement of other factors in the study, but, as the main objective of our study was to assess the changes in dietary habits and physical activity among diabetic patients pre- and during the pandemic, we have decided to remove the discussion of these associated factors from the result section to avoid ambiguity. We have carefully revised the result section to better align with the study's main objective and have taken into account the comments of both the reviewers and the editor.*

**Introduction**

The introduction require revision to strengthen understanding of the context involved.
Lines 46 - 50 should read; "Coronavirus disease 2019 (COVID- 19) was identified and disclosed for the first time in Wuhan, China, at the end of 2019, which was caused by "Severe Acute Respiratory Syndrome Coronavirus 2 (SARS CoV-2) viral infection, and rapidly spread around the globe [1]. In response to the rapid spread of the disease, governments around the world resolved to implement emergency measures such as lockdowns isolation, quarantine, and social and physical distancing to interrupt community transmission of the virus [2].

*We’ve modified the statements on lines 44-49 of the clean version as suggested.*

**Materials and methods:** Lines 122 to 130: Understanding the descriptions of the variables in the study would benefit greatly if they are organized as "Primary Outcome variable: Self-Reported Dietary Habits" and "Self-Reported Physical Activity" with descriptions of what constitutes each. And Secondary Outcome of "BMI" if measured see line 132.

*We’ve reorganized the variables as “Primary Outcome variable: “Self-reported Dietary Habits” and “Self-Reported Physical Activity” with descriptions on lines 133-142 of the clean version of revised manuscript. We’ve considered BMI as an independent variable. This is why we’ve not reorganized BMI as secondary outcome variable.*

**Statistical Analysis:** The focus of the study should guide the data analysis structure. There appears to be a drifting away from the main objectives for the study (see lines 96-99) "The aim of this study was to investigate the changes in dietary habits and physical activity of diabetic patients in Bangladesh during COVID-19 pandemic". Here the study in lines 147-151 is referring to associations instead of comparing pre-Covid-19 outcomes with during Covid-19 values at the time of the study. I believe that Analysis of Variance of Z-test would have been the most appropriate for comparing pre-pandemic dietary experiences and physical experiences with Covid-19 period, Clarity is required here applying the most appropriate statistical tools for comparison rather than correlation.

*Thank you for your valuable comments and suggestions regarding the analysis structure of our study. We agree that the focus of the study should guide the data analysis, and we appreciate your insight into the potential drifting away from the main objectives. According to your suggestion, we have re-analyzed the dataset and reported the changes in dietary pattern and physical activity along with its significance testing using the McNemar-Bowker test. McNemar-Bowker test is an appropriate statistical test to assess the changes in dietary pattern and physical activity pre- and during the COVID-19 pandemic period. The* *McNemar-Bowker Test is used for testing paired table symmetry. We believe that this approach aligns well with the objectives of our study and provides a clearer understanding of the impact of the COVID-19 pandemic on the lifestyle changes of the diabetic patients in Bangladesh.*

Lines 143 - 145 should read; "The sociodemographic characteristics and diabetes related variables were reported as frequency distribution while continuous variables of age, weight(kg), height (M) and BMI are expressed as mean and standard deviation.” Line 145 should read; "Changes in dietary pattern and physical activity before and during the pandemic were categorized as "increased", "decreased", and "No change" and scored as 2-points, 1-point and zero-point for each participant in the study.”

*Lines 143-145 are modified as suggested by the Editor and the reviewers on lines 153-155 of the clean version. We’ve reanalyzed the dataset to determine the changes in dietary pattern and physical activity. During the revision, the we didn't generate any new categorical variables. Therefore, we haven’t categorized as "increased", "decreased", and "No change" during the analysis. That is why, we’ve removed the line 145 of previous version in the clean version of the revised manuscript.*

**Results:**

The results in the manuscript needs to be organized to facilitate clarity and understanding. Line 221 "Figure 3" should be "Table". Table 2 is appearing twice and lines 179 and lines 189.
Table 3 comparing the pre-pandemic and during the pandemic is inaccurate for the table heading. What statistical tool was used to determine the p-values?

*We have carefully considered your feedback and have made the necessary revisions to improve the clarity of our results section. Specifically, we have changed "Figure 3" to "Table 3" and removed the duplicate Table 2. In addition, we have deleted the original Table 3, which determined the association of the level of physical activity with other independent variables among the study participants. We have generated a new Table 3 comparing the pre-pandemic dietary pattern with the COVID-19 period. To determine the p-values for this new table, we performed the McNemar-Bowker test.*

Authors' attention is urgently called to re-analyze Table 3 and use frequency data rather than percentages because these do not represent the data required to explain the table, especially if the statistical tool used is the Chi-square test of goodness-of-fit, which should be the most appropriate. The various categories do not add up to 100% for each response categories. If these variables were scored then apply accordingly.

*We understand that percentages may not have been the appropriate measure to explain the data in Table 3, especially when using the Chi-square test of goodness-of-fit. However, in the revised version of our manuscript, we have deleted the previous Table 3 and replaced it with a new one that compares the pre- and during pandemic dietary pattern. We have presented both frequency and percentage data in the revised Table 3 and used the McNemar-Bowker test to determine the p-values. We hope this approach adequately addresses the concerns raised by the reviewers regarding Table 3.*

Data analysis require a second review. Multinomial logistic regression is not clear for this study noting the objective of the study. If this analysis is relevant, then revise the title and objectives to reflect factors associated with changes in "...dietary and physical activity pre-pandemic and during the pandemic".

*Upon reviewing our analysis, we agreed that Multinomial logistic regression may not have been the most appropriate method to address our study objective. As suggested, we have re-analyzed the dataset focusing the study objective. We also deleted Table 4 during the revision, as it was irrelevant to our targeted objective and generated a new table presenting the changes in the level and duration of physical activity. We appreciate your suggestion to clarify our focus on investigating changes in dietary patterns and physical activity during COVID-19, rather than determining associated factors. To that end, we have re-analyzed the dataset and generated new versions of Table 3 and Table 4 to reflect our objective better.*

*We carefully and critically evaluated the reviewer’s comment and incorporated his/her suggested modifications in the manuscript. Detailed responses to the reviewer’s comments are provided below.*

Reviewers' comments:

**Comments to the Author**

**Reviewer #1:** There are few instances where some words are used interchangeably. There is a need for consistency. For instance, individuals recruited into quantitative study are referred to as respondents. While those recruited into qualitative study are referred to as participants
There are some statements that show no agreement between them. The authors should clarify this.

*Thank you for your feedback. We appreciate your input and have made the necessary changes to ensure consistency throughout the manuscript. We have replaced all instances of "participants" with "respondents" in the current study. We have also carefully reviewed the manuscript to ensure that no other words are interchangeably used.*

There is missing information in the methods on how data was collected.

*The data were collected by face-to-face interview and we added this on lines 117-118 of the clean version of revised manuscript . Additionally, we’ve modified method section based on the editor and reviewer’s comments so that it is more comprehensive.*

The results section needs a revisit.

*We re-analyzed the dataset and the result section has been modified accordingly. Specifically, original Table 3 and Table 4 have been removed and we generated new tables with required interpretations in the result section.*

The discussion and the conclusions need modification after the major issues in the result section are addressed.

*The discussion and the conclusions have been rewritten after the modified changes in the result section.*

Many typos and grammar errors

*Typos and grammar errors have been corrected by the authors.*

The attached comments will help the authors.

**Reviewer #2:** This study investigated the changes in dietary habits and physical activity of diabetic patients during the COVID-19 pandemic and compared the results to self-reported status at pre-pandemic. This would have been an excellent study if it was appropriately designed and implementation adequately articulated. The concept is excellent but there are serious flaws needing to be corrected to strengthen the manuscript. Below are my comments and suggestions for revision.

These comments are made based on the 1.) The title of the study, 2.) the objectives of the study and therefore, if authors’ decide to retain these two premise for the review would need to change the title and include modifications to the study objectives.

*We’ve retained the title and study objectives of our study and re-analyzed the data to address our study objectives as suggested by the reviewers*

The abstract to this study needs to be revised to communicate adequately what the study sought to achieve and what was found. The following represent revised suggestion. (See attached review for suggestions).

*Thank you for your feedback on the abstract of the study. According to your suggestion, we’ve revised the abstract to improve the communication of what the study sought to achieve and what was found.*

**Introduction**
The introduction require revision to strengthen understanding of the context involved. (See attached review for suggestions).

*We’ve modified the introduction as suggested by the reviewers and by the editor.*

**Materials and methods** require very careful appraisal of study design, participant enrolment into the study, instrument development and validity, data analysis.

*We have revised the material and methods section carefully considering the reviewer’s comment. We’ve detailed out the study design, data collection process, instrument development, validity, data analysis and management in the respective sections.*

**Results** The results in the manuscript needs to be organized to facilitate clarity and understanding. Line 221 "Figure 3" should be "Table". Table 2 is appearing twice and lines 179 and lines 189. Table 3 comparing the pre-pandemic and during the pandemic is inaccurate for the table heading. What statistical tool was used to determine the p-values?

*We have carefully considered your feedback and have made the necessary revisions to improve the clarity of our results section. Specifically, we have changed "Figure 3" to "Table 3" and removed the duplicate Table 2. In addition, we have deleted the original Table 3, which determined the association of the level of physical activity with other independent variables among the study participants. We have generated a new Table 3 comparing the pre-pandemic dietary pattern with the COVID-19 period. To determine the p-values for this new table, we performed a McNemar-Bowker test.*
